# Case Report: Real NPSLE? A patient with systemic sclerosis overlapping systemic lupus erythematosus presenting as epilepsy

**DOI:** 10.3389/fimmu.2023.1185501

**Published:** 2023-06-28

**Authors:** Jialin Zhang, Xiaodong Wu, Jing Xue, Lei Liu

**Affiliations:** ^1^ Department of Rheumatology, The Second Affiliated Hospital of Zhejiang University, School of Medicine, Hangzhou, Zhejiang, China; ^2^ Department of Teaching and Research, Jiande First People’s Hospital, Hangzhou, Zhejiang, China; ^3^ Department of Cardiology, The Second Affiliated Hospital of Zhejiang University, School of Medicine, Hangzhou, Zhejiang, China

**Keywords:** scleroderma renal crisis, neuropsychiatric systemic lupus erythematosus, glucocorticoid, systemic sclerosis, rheumatic immune diseases

## Abstract

Neuropsychiatric systemic lupus erythematosus (NPSLE) is the diagnosis that rheumatologists most often need to consider when a patient with lupus presents with neurologic symptoms. However, neurological involvement is rare in systemic sclerosis (SSc), and high doses of steroids tend to trigger scleroderma renal crisis (SRC). When a patient with SSc overlapping SLE presents with epilepsy and renal crisis, the exact diagnosis and whether to initiate high-dose glucocorticoid therapy are questions to ponder. Here, we report a patient with overlap syndrome (SSc overlapping SLE), who developed CNS symptoms, and improved after treatment against SRC after excluding NPSLE. We report this case with the aim of arousing the attention of rheumatologists to SSc and SRC-related encephalopathy when SSc was overlapped with SLE.

## Highlights

- NPSLE is not the only answer to CNS symptoms in patients with SLE overlapping SSc.
- Distinguishing between RPLS due to SRC and NPSLE is critical for the patient’s prognosis.

A 17-year-old female patient with a one-and-a-half-year history of SSc and SLE was admitted to our rheumatology department. Two days earlier, the patient had a sudden loss of consciousness with two seizures and the highest locally obtainable blood pressure reached 180/120 mmHg. Previously, she received 11 cycles of cyclophosphamide therapy with a cumulative dose of 6.6 g over the first 8 months of her disease, and prednisone was tapered from 20 mg/d at the time of initiation to 10 mg/d. The patient’s skin remained progressively sclerotic with severe Raynaud’s phenomenon in both hands, and fortunately she did not have obvious interstitial fibrosis in her lungs. Thereafter, the treatment regimen was changed to tocilizumab at 320 mg once a month, with the last administration of tocilizumab one month prior to this admission (totally three times).

**Figure 1:**
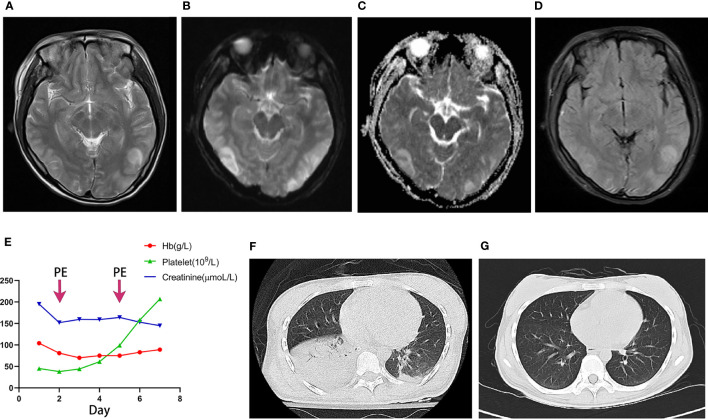
**(A–D)** MRI of the patient’s brain, multiple lesions in the bilateral frontoparieto-occipitotemporal lobe and left basal ganglia region: **(A)** T2 weighted image; **(B)** Diffusion Weighted Imaging (DWI); **(C)** Apparent Diffusion Coefficient (ADC); **(D)** Fluidattenuated Inversion Recovery (FLAIR). **(E)** Changes in hemoglobin, platelets and creatinine from day 1 to day 7, with the arrows referring to the time of plasma exchange therapy. **(F)** HRCT of the patient's chest at the time of admission. **(G)** Chest HRCT on day 7.

Her blood pressure was 153/128 mmHg and the heart rate was 110 bpm. She was confused, unconscious and in a drowsy state (having been treated with midazolam). Further investigation revealed serum creatinine 152.0 μmol/L (eGFR 33.8 ml/min), hemoglobin 81 g/L, platelet count 38×10^9^/L, C reactive protein (CRP) 58.1 mg/L, erythrocyte sedimentation rate (ESR) 80.0 mm/h, brain natriuretic peptide (BNP) 5542 pg/m, interleukin (IL)-6 1059 pg/mL. The titer of ANA was 1:160, and anti-SSA, Ro-52, SSB, Scl-70 and centromere antibodies were positive. The complement levels were normal and the SLEDAI score was 5. Enhanced MRI of the head suggested reversible posterior leukoencephalopathy (RPLS) (**Figures 1A–D**). Chest CT indicated exudation in both lungs (**Figure 1F**). Lumbar puncture was performed, and cerebrospinal fluid (CSF) pressure of 310 mmH_2_O was measured. CSF biochemistry suggested a protein level of 127.3 mg/dL, and the nucleated cell count was 0. Novel cryptococcal smear and G+GM test of the CSF were negative.

The patient had hypertension and progressive deterioration of renal function, as well as a significant decrease in hemoglobin and platelets. The diagnosis of SRC was clear, and there was a possibility of thrombotic microangiopathy (TMA). With the history of SLE and the absence of signs of infection on cerebrospinal fluid tests, NPSLE needed to be considered, and initiation of high-dose glucocorticoid therapy was imminent. However, the patient suffered from SRC, and steroid therapy was highly likely to aggravate it. The apparently elevated intracranial pressure became a key to deciphering the problem. Intracranial pressure in patients with NPSLE is generally normal or mildly elevated, and the intracranial pressure of 320 cmH_2_0 was abnormal. And the RPLS was more frequent in hypertension than NPSLE. We believe that RPLS due to SRC-related hypertension was more likely the cause of epilepsy other than NPSLE. Therefore, we started captopril 25 mg Q6h and plasma exchange therapy along with prednisone (20 mg/d).

On the second day, the patient became conscious and did not have further seizures. The hemoglobin and platelets gradually increased and creatinine decreased (**Figure 1E**). On the seventh day, the patient was fully conscious, with blood pressure 110/76mmHg. The chest CT showed that the exudate in both lungs was significantly absorbed (**Figure 1G**).

Central nervous system involvement is rare in SSc. When patients with SSc overlapping SLE present with neurological symptoms, rheumatologists tend to consider NPSLE first. The treatment of NPSLE requires high doses of glucocorticoids, however, glucocorticoids, particularly at high dose (> 15 mg/day), have long been associated with development of SRC (1). Nevertheless, it is difficult to exclude NPSLE based only on clinical and radiographic signs; after all, RPLS is also one of the manifestations of NPSLE (2, 3). Based on the abnormally high intracranial pressure in this patient, we considered that her CNS symptoms were caused by SRC-Hypertensive Encephalopathy induced RPLS, and that lowering the blood pressure and treatment for SRC was the key rather than high dose of glucocorticoids. The low activity of SLE and the rapid improvement after captopril treatment allowed us to confirm our assumptions. Therefore, when we encounter patients with SSc overlapping SLE presenting with encephalopathy, we must be aware of blood pressure and intracranial pressure to distinguish SRC-associated RPLS, and effective antihypertensive therapy will quickly improve patient consciousness and corroborate our diagnosis. We report this case with the aim of arousing the attention of rheumatologists to SSc and SRC-related encephalopathy when SSc was overlapped with SLE.

## Data availability statement

The original contributions presented in the study are included in the article/supplementary material. Further inquiries can be directed to the corresponding author.

## Ethics statement

The studies involving human participants were reviewed and approved by Ethics Committee of the Department of the Second Affiliated Hospital of Zhejiang University, School of Medicine. Written informed consent to participate in this study was provided by the participants’ legal guardian/next of kin. Written informed consent was obtained from the participant/patient(s) for the publication of this case report.

## Author contributions

JZ and XW performed data analysis and wrote the paper. LL and JX provided clinical information and supervised the study. All authors contributed to the article and approved the submitted version.
